# Four cases of endometrioid borderline ovarian tumour: case reports and literature review

**DOI:** 10.1259/bjrcr.20170062

**Published:** 2017-10-21

**Authors:** Eriko Nakagawa, Kaoru Abiko, Aki Kido, Sachiko Kitamura, Ken Yamaguchi, Tsukasa Baba, Sachiko Minamiguchi, Noriomi Matsumura

**Affiliations:** ^1^Department of Gynecology and Obstetrics, Kyoto University Graduate School of Medicine, Kyoto, Japan; ^2^Department of Diagnostic Imaging and Nuclear Medicine, Kyoto University Graduate School of Medicine, Kyoto, Japan; ^3^Department of Diagnostic Pathology, Kyoto University Graduate School of Medicine, Kyoto, Japan

## Abstract

Endometrioid borderline tumours (EBTs) of the ovary are uncommon tumours of low malignant potential. They consist of atypical endometrioid cells lacking destructive stromal invasion. As the prognosis of EBT is excellent, excessive treatment should be avoided and preoperative diagnosis is important. Here we report four cases of ovarian EBTs along with imaging findings and a review of literature. The average patient age was 52 years. They presented with abdominal discomfort or abnormal vaginal bleeding. The final diagnoses for all four cases were EBT Stage IA with endometriosis. Pathologically, one case was an adenofibromatous type tumour, and three cases were intracystic type tumours. Two patients had concurrent endometrial cancer. MRI of the tumours showed enhanced solid components. The intracystic type tumours presented a dendritic structure in the cyst; fine papillary branches surrounded a low-signal trunk on *T*_2_ weighted imaging. Positron emission tomography demonstrated marked fluorodeoxyglucose uptake in the solid component. One case with MRI 3 years before diagnosis indicated that the tumour arose in ovarian endometriotic cyst. EBT cases were difficult to distinguish from malignant ovarian tumours preoperatively. Intraoperative frozen section analysis may aid to determine treatment. Prognoses were excellent. Care should be taken for co-existing endometrial cancer.

## Introduction

Endometrioid borderline tumours (EBTs) of the ovary are defined as tumours of borderline malignancy, which consist of atypical endometrioid epithelium but lack destructive stromal invasion.^1^ The incidence of EBTs is very low, comprising up to 0.2% of all epithelial ovarian tumours.^2^ EBTs are often diagnosed at an early stage with a good prognosis. Here we report four cases of EBTs diagnosed at our institution.

## Case review

### Case 1

**Figure 1.**
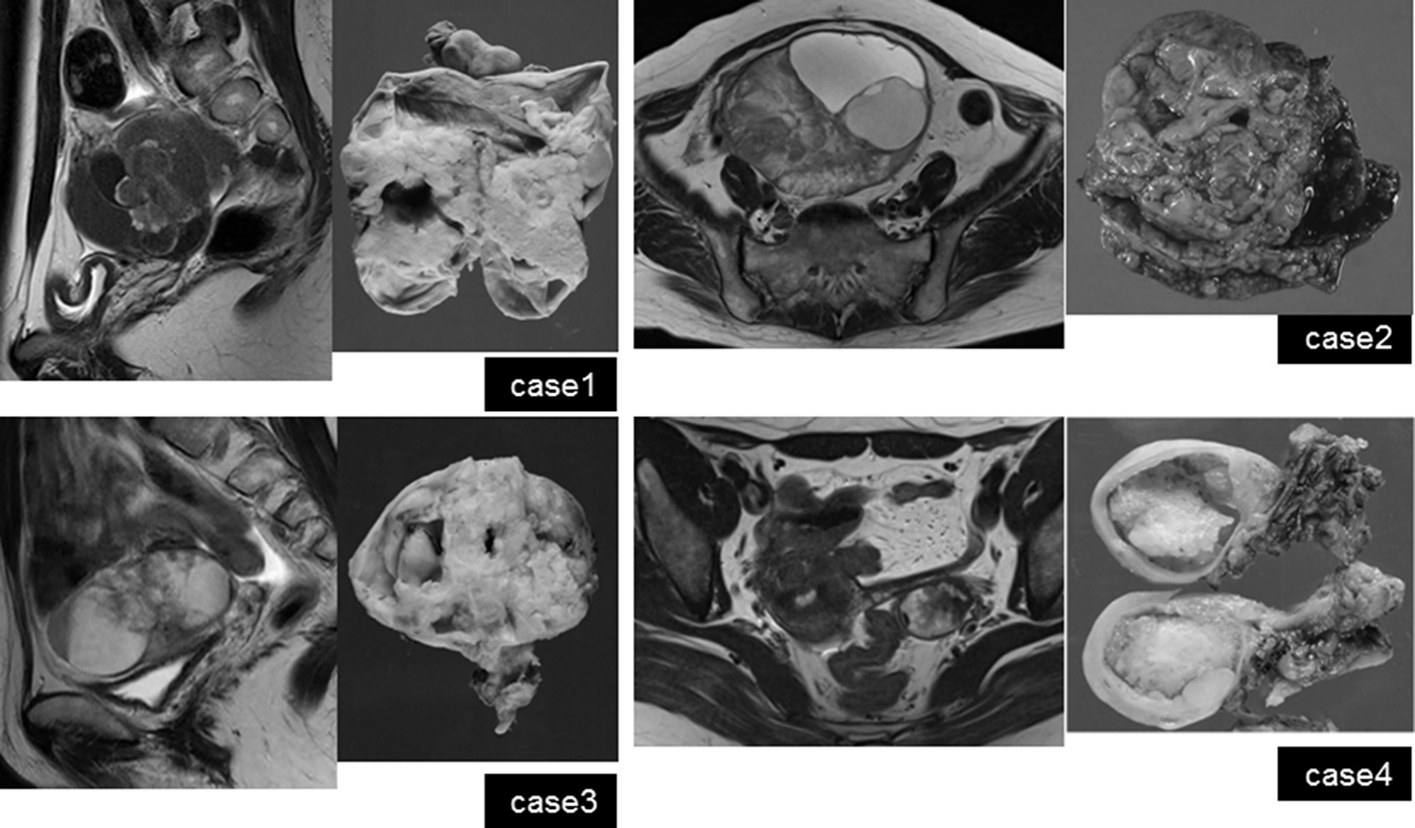
Imaging and macroscopic findings of case 1–4. *T*_2_WI (Cases 1–4) shows the tumours consisting of cystic and solid lesions. Reflecting abundant stroma, the components are of low signal intensity, and are of various shapes, including papillary projections on T2WI (Cases 2–4). Together with the high intensity on T_1_WI (data not shown), multicystic portions are assumed to have bloody content, but show variable intensity. The MR images are reminiscent of malignant tumours arising from endometriosis such as endometrioid carcinoma or clear cell carcinoma. T1WI, *T*_1_ weighted image; T2WI, *T*_2_ weighted image.

**Figure 2.**
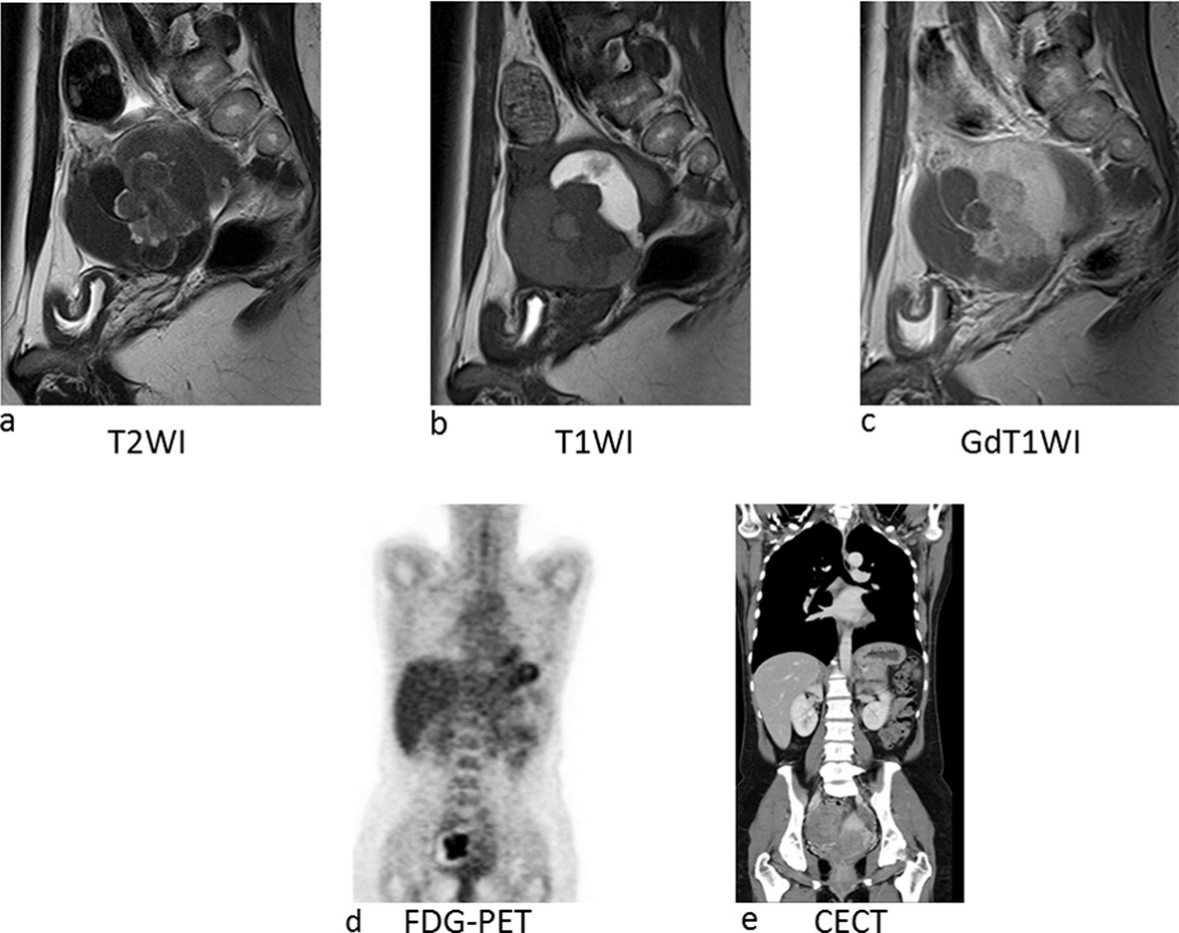
Imaging of Case 1. (a) Sagittal *T*_2_ weighted image shows multicystic tumour. Each locule shows different signal intensity and also include papillary lesion in the middle of the tumour. (b) On sagittal *T*_1_ weighted image, some of the locules showed high signal intensity, indicating haemorrhagic content within the cyst. (c) Gadolinium enhanced *T*_1_ weighted image, middle part of the locules and papillary lesion within the locule were well enhanced and indicated the presence of solid component. (d) FDG-PET image shows strong uptake with irregular shape at the right side of the pelvis. According to the contrast enhanced CT image (e), those uptake corresponds to the right ovarian tumour, especially, solid component within the tumour. FDG-PET, fluorodeoxyglucose positron emission tomography.

**Figure 3.**
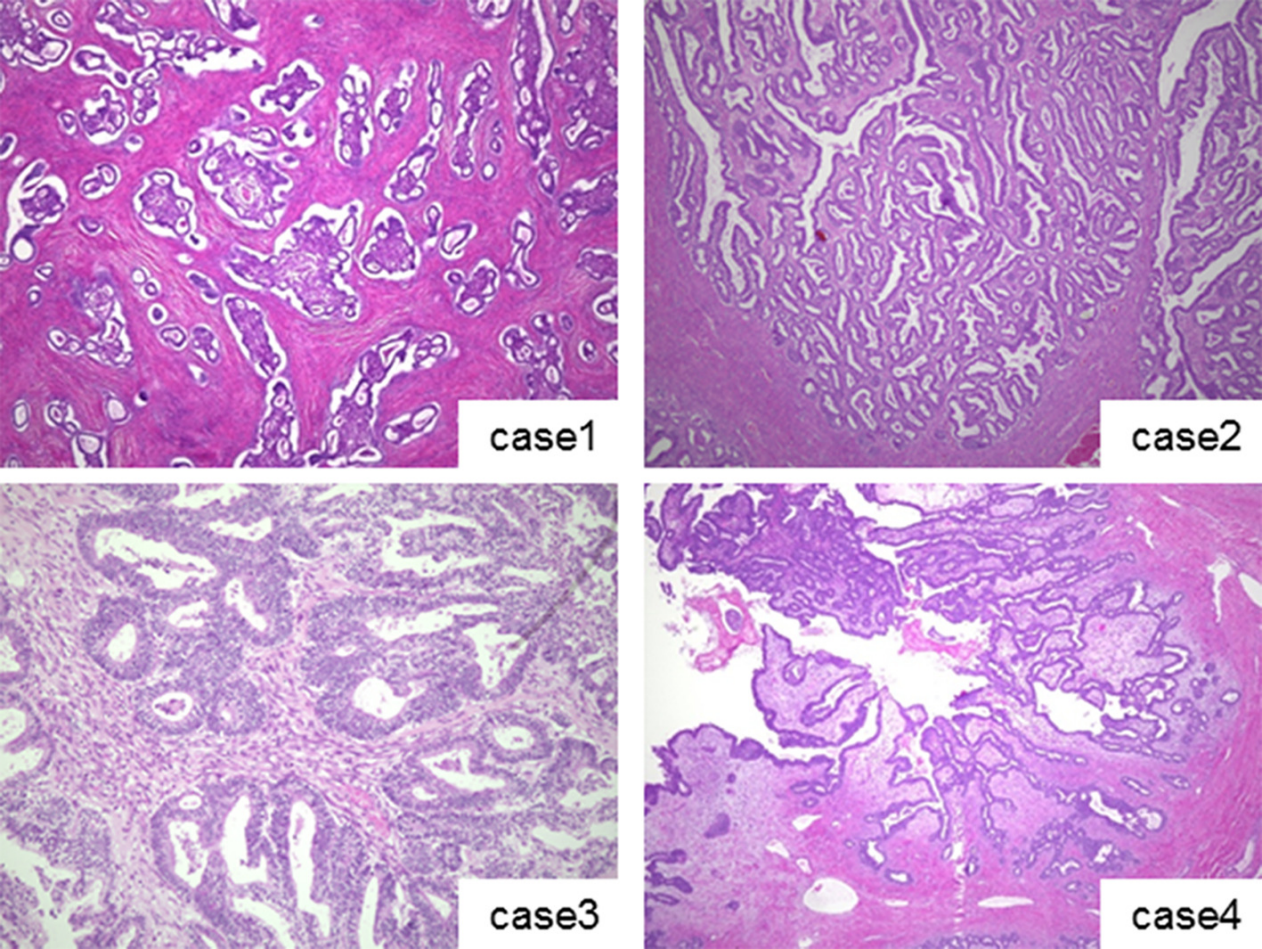
Microscopic images of endometrioid borderline tumours (EBT) of case 1–4 stained with hematoxylin and eosin. Case 1, adenofibromatous type EBT. Islands of atypical endometrioid glands are proliferating in fibrous stroma. Case 2, intracystic type EBT. Papillary proliferation of atypical endometrioid epithelium is noted in the cyst. Case 3, intracystic type EBT. Papillary and glandular proliferation of atypical columnar endometrioid cells are noted. An area with fusion of crowded glands is visible but does not exceed 5 mm in size, and lacks destructive stromal invasion. Case 4, intracystic type EBT. Note papillary growth of atypical endometrioid cells.

The patient was a 47-year-old, gravida 2, para 2 female. She presented with lower right abdominal pain. MRI revealed a complex solid and cystic lesion measuring 9 cm in the right ovary (Figure 1, upper left; Figure 2a–c). Some of the cystic locules showed high signal intensity on *T*_1_ weighted image (WI), indicating haemorrhage. A large enhanced solid component showed marked fluorodeoxyglucose (FDG) uptake on positron emission tomography (PET) (Figure 2d). A malignant ovarian tumour was suspected, so she underwent laparotomy. Analysis of an intraoperative frozen section of the tumour suggested a malignant or borderline endometrioid tumour of the ovary. During surgery, peritoneal thickening and adhesion in the Douglas pouch were observed, indicating pelvic endometriosis. Analysis of formalin-fixed paraffin embedded (FFPE) specimens revealed islands of endometrioid tumour cells showing moderate cytologic atypia proliferating within dense fibrous stroma (Figure 3, upper left). The case was diagnosed as EBT, pT1aN0M0.

### Case 2

**Figure 4.**
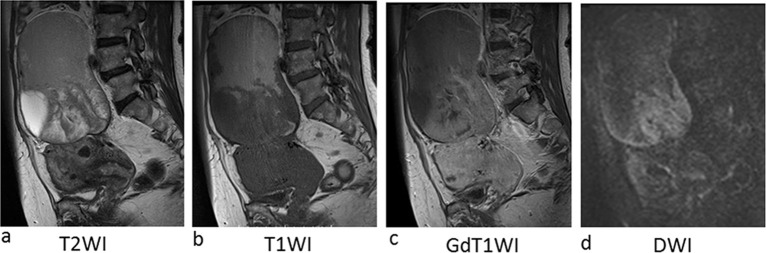
Imaging of Case 2. (a) On sagittal T_2_ WI, large cystic tumour is located above the uterus. Cystic lesion and papillary projection mixing low and high signal intensity were observed within the cyst. (b) On sagittal T1WI, upper half of the tumour shows high signal intensity, indicating haemorrhagic content. The rest of the lesion showing low signal intensity on T1WI were enhanced on gadolinium enhanced T1 WI (c). Enhnaced lesion shows high signal intensity on diffusion weighted image (d). T1WI, *T*_1_ weighted image; T2WI, *T*_2_ weighted image.

The patient was a 65-year-old, gravida 2, para 2 post- menopausal female. She presented with vaginal bleeding. She was pointed out a pelvic mass on clinical examination and ultrasound. MRI showed a multiloculated mass measuring 15 cm of mixed high and low signal intensities on T1WI. Within a cyst, irregular shaped papillary projections were observed at the tumour wall on T2WI and were well enhanced indicating solid components (Figure 1 upper right, Figure 4a-c). This lesion showed restricted water diffusion on diffusion weighted imaging (DWI) (Figure 4d). A malignant ovarian tumour was suspected, and laparotomy was performed. A large right ovarian tumour was found extending into the retroperitoneum, strongly adherent to the pelvic wall. Analysis of intraoperative frozen sections revealed EBT. Final pathological diagnosis was EBT, pT1aN0M0. Intracystic papillary proliferation of atypical endometrioid epithelium was observed (Figure 3, upper right), with coincidental non-atypical endometriosis. A benign endometrial polyp was detected in the uterine endometrium.

### Case 3

**Figure 5.**
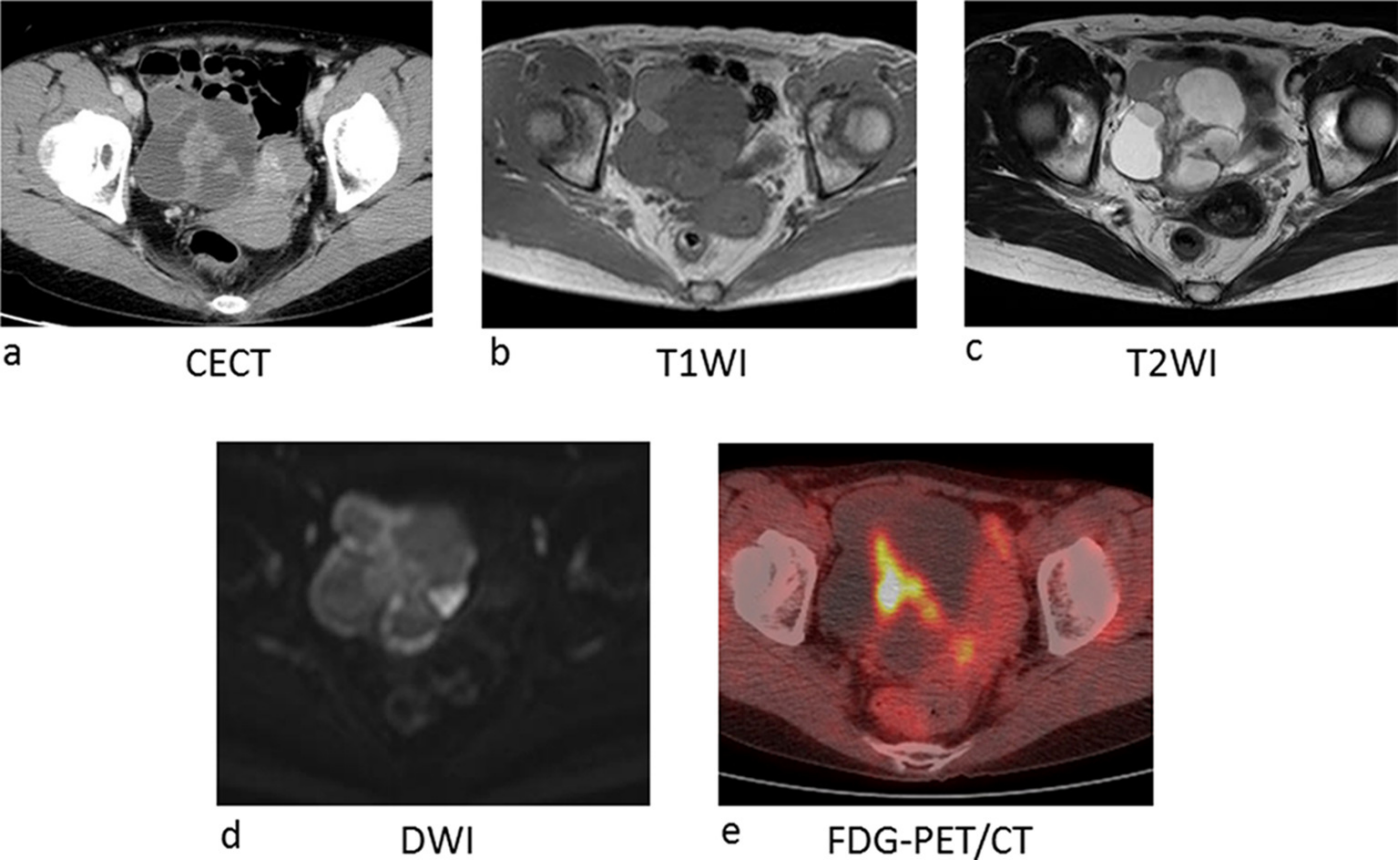
Imaging of Case 3. (a) On contrast enhanced CT image, complex solid and cystic tumour is located at the right side of the uterus. On both *T*_1_ (b) and *T*_2_ weighted image (c), each locule shows different signal intensities with shading due to haemorrhage within the cyst. Papillary lesion was observed on the tumour septum on *T*_2_WI and enhanced on post contrast CT and higher signal intensity on Diffusion weighted image (d). (e) PET-CT shows strong uptake to the solid component. PET-CT, positron emission tomography CT.

The patient was a 54-year-old, gravida 2, para 2 post-menopausal female. She presented with lower abdominal discomfort and a right ovarian mass was pointed out by pelvic examination. Endometrial cytology detected adenocarcinoma cells. MRI and CT revealed a complex solid and cystic lesion of 8.4 cm diameter within the right ovary. A papillary lesion was observed on the tumour septum on T2WI and enhanced on post-contrast CT (Figure 1 lower left, Figure 5a-c). PET-CT showed strong uptake within the solid component (Figure 5d,e). Endometrial lesions were indistinct on imaging studies including CT and MRI. Concurrent ovarian and endometrial cancer was suspected, and she underwent surgery. During the surgery, the multiloculated right ovary measuring 8 cm was found to be adherent to the pelvic peritoneum. Blueberry spots, or blue tiny spots on peritoneum were observed in the Douglas pouch, indicating pelvic endometriosis. Intraoperative frozen section analysis suggested EBT. Analysis of FFPE specimens revealed an intracystic proliferation of atypical endometrioid epithelium showing crowded fusing glands (Figure 3 lower left). The area of confluent glands were smaller than 5 mm, and destructive stromal invasion was absent, justifying the diagnosis of EBT, pT1aN0M0. Coincidental endometrioid carcinoma, Grade 1, pT1aN0M0 was found in the uterine endometrium.

### Case 4

**Figure 6.**
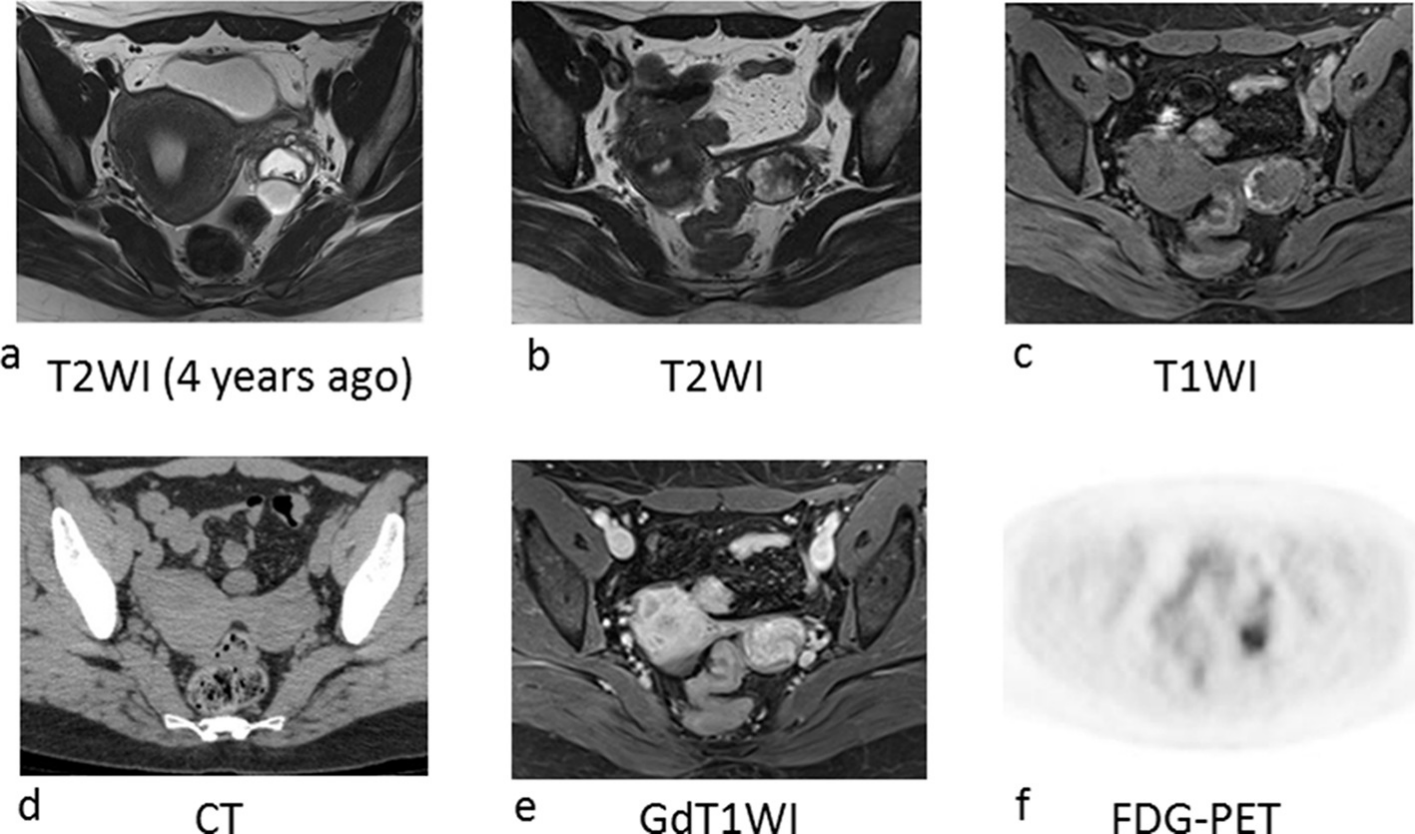
Imaging of Case 4. (a) MRI 4 years before the operation. The content of the cyst in the left ovary showed high intensity on T2WI with *T*_2_ dark spot sign and intermediate-to-low intensity on T1WI (not shown) indicating endometriotic cyst. (b) MRI 1 month before the operation. On axial T2WI, small tumour is observed at the left side of the uterus. There was papillary projections with low signal intensity were observed within the cyst. Haemorrhage is observed at the peripheral of the tumour on T1WI (c) and CT (d). Papillary projections within the cyst were well enhanced on gadolinium enhanced T1WI (e) and shows strong uptake at FDG-PET (f). FDG-PET, fluorodeoxyglucose positron emission tomography; T1WI, *T*_1_ weighted image; T2WI, *T*_2_ weighted image.

The patient was a 41-year-old, gravida 0 female. She was diagnosed with a left ovarian endometriotic cyst and uterine myomas when she was 34-years-old. At the age of 36, she was diagnosed with breast cancer and underwent left mastectomy and axillary lymphadenectomy, chemotherapy, and radiotherapy. She then commenced gonadotropin-releasing hormone agonist and tamoxifen. MRI at the age of 37 revealed an endometriotic cyst in her left ovary; the cyst showing high intensity on T2WI and intermediate-to-low intensity on T1WI without any solid component (Figure 6a). At the age of 40, she experienced intermittent vaginal bleeding lasting 6 months. Endometrial biopsy under hysteroscopy revealed endometrioid carcinoma, Grade 1. On this occasion the left ovarian tumour was unchanged in size when compared to the previous MRI scan, but there was now solid areas with strong enhancement within the haemorrhagic cyst on MRI (Figure 1 lower right, Figure 6b-e). Those solid lesions showed restricted water diffusion. Endometrial lesion was not recognized on MRI. PET-CT demonstrated FDG uptake in the solid portion of the left ovarian tumour (Figure 6f). A malignant left ovarian tumour with endometrial cancer was suspected. She underwent surgery. The 3-cm left ovarian cyst was adherent to the peritoneum. Intraoperative frozen section analysis demonstrated a seromucinous borderline tumour. In FFPE specimens, a left ovarian cyst was identified, with intracystic proliferation of papillary endometrioid epithelium with cellular and structural atypia (Figure 3, lower right). The case was diagnosed as EBT of the ovary, pT1aN0M0 and endometrioid carcinoma Grade 1 of the uterine endometrium, pT1aN0M0.

Table 1 lists patient characteristics, image study findings and pathological findings. Table 2 lists the results of immunohistochemistry for estrogen receptor, p53, and ARID1A. None of the patients underwent adjuvant therapy, but they were all well without recurrence. All patients provided written informed consent.

**Table 1.** Clinicopathologic characteristics and imaging findings of the four cases of EBT

	Age	Symptom	Hormonal status	Size (cm)	Stage	Frozen section report	Endometriosis	Endometrial lesion	Dendric structure on MRI	FDG uptake on PET-CT
1	47	Abdominal pain	Pre- menopausal	9	IA	EBT or EC	+	none	-	+
2	65	Vaginal bleeding	Post- menopausal	13	IA	EBT	+	Polyp	+	Not done
3	54	Abdominal discomfort	Post- menopausal	8	IA	EBT	+	EC	+	+
4	41	Vaginal bleeding	TAM intake	3	IA	SMBT	+	EC	+	+

EBT, endometrioid borderline tumour; EC; endometrioid carcinoma; FDG, fluorodeoxyglucose ; PET-CT, positron emission tomography CT; SMBT, seromucinous borderline tumour; TAM; tamoxifen. Clinical findings, characteristics in imaging are shown.

**Table 2.** Immunohistochemistry findings of the four cases of EBT

	ER	p53	ARID1A
1	+	WT	WT
2	+	WT	WT
3	+	WT	WT
4	+	WT	WT

ARID1A, AT-rich interactive domain-containing protein 1A; ER, estrogen receptor, WT, wild type.

Table 3 lists 122 reported EBT cases in the literature. Case reviews reporting more than 3 cases are included.^2–5^

**Table 3.** Clinicopathologic review of the reported cases of EBT

Reports	Adenofibromatous	Papillary	Endometriosis	Endometrial lesion	Hysterectomy	Relapse
Bell (2000) 41 cases	25 (61.0%)	16 (39.0%)	14 (34.1%)	*	*	0
Snyder (1988) 31 cases	12 (38.7%)	19 (61.3%)	16 (51.6%)	AEH 4 EP 3 HWOA 5	16 (51.6%)	0
Roth (2003) 30 cases	14 (46.7%)	16 (53.3%)	19 (63.3%)	7	18 (60.0%)	0
Uzan (2012) 16 cases	*	*	3 (18.8%)	1	5 (31.3%)	1
Present report 4 cases	1 (25%)	3 (75%)	4 (100%)	EC2 EP1	4 (100%)	0

AEH, atypical endometrial hyperplasia; EBT, endometrioid borderline tumour; EC, endometrioid carcinoma; EP, endometrial polyp; HWOA, hyperplasia without atypia. Reported EBT case series in the literature of more than four cases are shown.

## Discussion

Pathologically, EBT is classified into two types: adenofibromatous type, with atypical endometrioid glands proliferating among abundant stroma, and intracystic type with proliferation of atypical endometrioid glands inside a cyst.^1^ In the present case report, one adenofibromatous (Case 1) and three intracystic (Cases 2–4) types were described (Table 1). As both types of EBT show prominent solid growth, the differential diagnosis includes malignant ovarian tumours. For a diagnosis of EBT, confluent gland proliferation must not exceed 5 mm, and destructive stromal invasion must be lacking on histological examination, otherwise it is an obvious malignant tumour.^1^ Clinically, EBT is a tumour with excellent prognosis. The majority of the reported cases are confined to a unilateral ovary, and metastasis and recurrence are rare (Table 3).^3–6^

EBT is often associated with endometriosis and endometrial lesions. Among 122 EBT cases, 56 (46%), including 4 cases from our institution, were associated with endometriosis (Table 3).^3–6^ Among 43 patients who underwent hysterectomy, endometrial lesions including endometrial polyps, endometrial hyperplasia, and endometrial cancer were diagnosed in 23 patients (53%) (Table 3). In the four patients from our institution, one had endometrial polyp and two had early-stage endometrial cancer (Table 1). Case 4, with endometrial cancer, had a history of tamoxifen intake, suggesting a role for tamoxifen in tumorigenesis from orthotopic and ectopic endometrium.^7^

On a comprehensive search of the literature, there are no reports dedicated to MRI findings for EBT tumour types. MRI visualization of the four cases presented here showed mixed tumours with cystic and solid portions (Figure 1). The solid portions demonstrated enhancement and water diffusion was restricted, indicating malignant tumours. In Case 1, which was pathologically diagnosed as adenofibromatous type EBT, abundant stroma in histological examination might be reflected in low-signal intensity in the solid portion on *T*_2_ WI. In Cases 2–4, which were intracystic type EBTs, showed a thickened stroma corresponding to low-signal intensity on *T*_2_ WI in the center of the solid portion, and at the periphery, fine branch-like structures of low-signal intensity were observed. MRI findings of the intracystic type EBTs were similar to serous borderline tumour (SBT) of the ovary,^8^ but reflecting abundant stroma characteristic in EBT histology, low-intensity portion on *T*_2_ WI was thicker than that in SBT. Multicystic portions showed stained-glass patterns with various intensities. Case 4 showed a solid portion in a single cyst. In this case, MRI taken 3 years prior to diagnosis showed endometriotic cyst of the ovary, indicating that the tumour arose in endometriosis tissue. Three patients in the current study underwent PET-CT, and all three showed marked FDG uptake in the solid portion of the tumour (Table 1, Figures 2,5,6). Studies with more cases are required to clearly define PET characteristics of EBT.

Intraoperative consultation based on the pathology of frozen sections of tumour may aid in deciding adequate operative procedures for the ovarian tumour.^9^ In the four cases presented here, 3 were diagnosed as EBT, and 1 was diagnosed as seromucinous borderline tumour (Case 4). However, in Case 1 the pathologist stated that endometrioid carcinoma could not be excluded. Cross et al reported that in their case series of 22 endometrioid tumours, 19 cases that were diagnosed as borderline tumours by intraoperative frozen section analysis were ultimately diagnosed as malignant tumours.^10^ This data suggests that endometrioid tumours tend to be under-diagnosed. Because of the high suspicion for malignant tumours based on preoperative imaging studies we performed staging laparotomy including lymphadenectomy in all four cases. As EBTs are very rare, more research with larger case series is needed to determine the reliability of frozen section analyses.

## Conclusions

We reported four cases of EBT along with a review of the literature.

Imaging studies of our four cases of EBT presented mixed solid and cystic lesions, often including haemorrhagic cysts, with papillary solid projections demonstrating low signal intensity on T2WI. Enhancement, water restriction, and FDG uptake were observed in the solid components, making the cases almost indistinguishable from malignant ovarian lesions. Intraoperative frozen section analysis may help determine treatment. Concurrent endometrial lesions including endometrial cancer need to be considered and excluded.

## Learning points

1. MRI in EBT of the ovary demonstrated heterogeneous tumours containing both solid and cystic components. The solid components demonstrated enhancement and water restriction, making them difficult to distinguish from malignant tumours.
2. Many EBT cases are accompanied with endometriosis and some arise in endometriotic cysts.
3. Concurrent endometrial lesions are frequently encountered, especially endometrial cancer.

## Consent

Written informed consent for the case to be published (including images, case history and data) was obtained from the patient(s) for publication of this case report, including accompanying images.

## References

[1] Kurman RJ, Carcangiu ML, Herrington CS, Young RH. (eds) WHO Classification of Tumors of Female Reproductive Organs, 4th edn Lyon: International Agency for Research on Cancer, 2014.

[2] Ellenson LH, Carinelli SG, Cho KR, Kim KR, Kupryjanczyk J, Prat J et al Endometrioid tumors : Kurman RJ, Carcangiu ML, Herrington CS, Young RH, WHO Classification of Tumors of Female Reproductive Organs. 4th edn. Lyon, France: International Agency for Research on Cancer; 2014.

[3] Bell KA, Kurman RJ. A clinicopathologic analysis of atypical proliferative (borderline) tumors and well-differentiated endometrioid adenocarcinomas of the ovary. Am J Surg Pathol 2000; 24: 1465–79. 11075848 10.1097/00000478-200011000-00002

[4] Roth LM, Emerson RE, Ulbright TM. Ovarian endometrioid tumors of low malignant potential: a clinicopathologic study of 30 cases with comparison to well-differentiated endometrioid adenocarcinoma. Am J Surg Pathol 2003; 27: 1253–9. 12960810 10.1097/00000478-200309000-00009

[5] Snyder RR, Norris HJ, Tavassoli F. Endometrioid proliferative and low malignant potential tumors of the ovary. A clinicopathologic study of 46 cases. Am J Surg Pathol 1988; 12: 661–71. 3414893 10.1097/00000478-198809000-00002

[6] Uzan C, Berretta R, Rolla M, Gouy S, Fauvet R, Darai E, et al Management and prognosis of endometrioid borderline tumors of the ovary. Surg Oncol 2012; 21: 178–84. 22418038 10.1016/j.suronc.2012.02.002

[7] McCluggage WG, Bryson C, Lamki H, Boyle DD Benign, borderline, and malignant endometrioid neoplasia arising in endometriosis in association with tamoxifen therapy. Int J Gynecol Pathol 2000; 19: 276–9. 10907178 10.1097/00004347-200007000-00013

[8] Tanaka YO, Okada S, Satoh T, Matsumoto K, Oki A, Nishida M, et al Ovarian serous surface papillary borderline tumors form sea anemone-like masses. J Magn Reson Imaging 2011; 33: 633–40. 21287653 10.1002/jmri.22430

[9] Ratnavelu ND, Brown AP, Mallett S, Scholten RJ, Patel A, Founta C, et al Intraoperative frozen section analysis for the diagnosis of early stage ovarian cancer in suspicious pelvic masses. Cochrane Database Syst Rev 2016; 3: CD010360. 26930463 10.1002/14651858.CD010360.pub2 PMC6457848

[10] Cross PA, Naik R, Patel A, Nayar AG, Hemming JD, Williamson SL, et al Intra-operative frozen section analysis for suspected early-stage ovarian cancer: 11 years of Gateshead Cancer Centre experience. BJOG 2012; 119: 194–201. 21895958 10.1111/j.1471-0528.2011.03129.x

